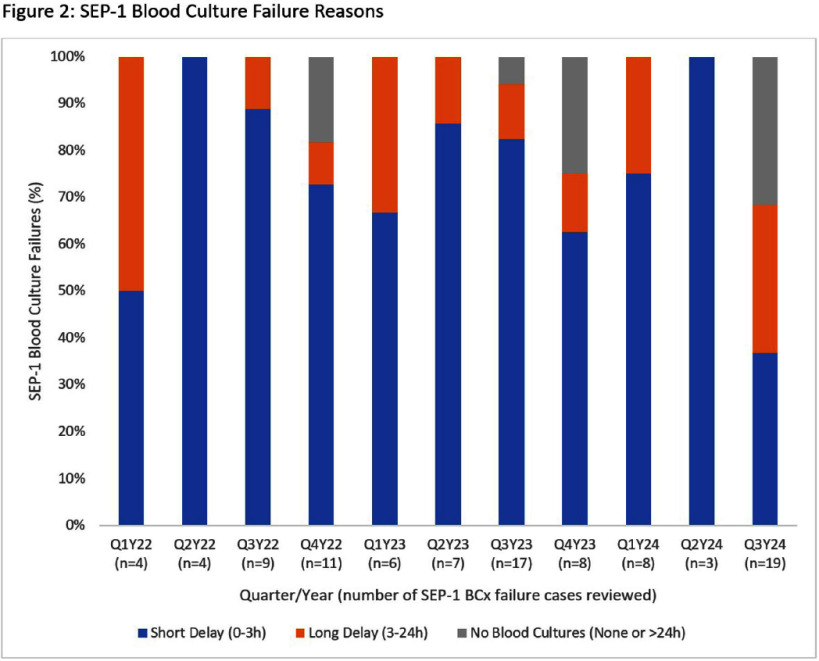# Are SEP-1 and Blood Culture Stewardship at Odds? Retrospective Review of SEP-1 Failures Pre- and During Blood Culture Shortage

**DOI:** 10.1017/ash.2025.220

**Published:** 2025-09-24

**Authors:** Jonathan Ryder, Trevor Van Schooneveld, Cynthia Japp, Kelly Cawcutt

**Affiliations:** 1 University of Nebraska Medical Center; 2 Nebraska Medicine

## Abstract

**Background:** In summer 2024, our institution implemented enhanced blood culture (BCx) stewardship due to a global BCx shortage. Centers for Medicare and Medicaid Services’ (CMS) SEP-1 quality measure requires BCx. We evaluated whether the BCx shortage and stewardship impacted SEP-1 compliance. **Methods:** A retrospective review of SEP-1 data from an academic and a community hospital (1/1/2022-9/30/24) was performed and grouped by quarter. SEP-1 compliance was abstracted per CMS criteria. Failures due to BCx were categorized by timing of antibiotics prior to BCx: short delay (0-3 hours after antibiotics), long delay (>3 to 24 hours or no BCx). SEP-1 failures due to no BCx underwent manual chart review to determine infectious source, alternative culture obtainment, and adjudication if BCx would have changed clinical management. BCx failures were compared descriptively by quarter with emphasis on the BCx shortage (quarter 3 of 2024). **Results:** Over 11 quarters, 690 cases were abstracted for SEP-1 review (mean 62.7 cases/quarter). The mean SEP-1 success rate pre-shortage was 51.1%, while SEP-1 success rate during BCx shortage was 40.3%, the lowest rate since 2022. SEP-1 failures due to improper BCx obtainment were the cause of SEP-1 failure in a mean of 12.3% of cases reviewed pre-shortage, but this increased to 30.6% during the BCx shortage (Figure 1). Pre-shortage BCx failures were due to short delays (77.9%), long delays (15.6%), and no BCx (6.5%). During the shortage, BCx failures were more commonly due to long delays (31.6%) or no BCx obtained (31.6%) (Figure 2). The majority (7/12) of cases with no BCx obtained were during the shortage. Upon adjudication of no BCx cases, positive BCx would have significantly changed management in only 2/12 cases. Reasons BCx would not have changed management included urinary tract infection with positive urine culture (5/12), non-infectious/non-bacteremic diagnoses (3/12), and community-acquired peritonitis with surgery (2/12). Specifically during the shortage, 5 were UTIs (4 had positive cultures), 1 with peritonitis (positive intra-abdominal cultures), and 1 without bacterial infection. **Conclusion:** SEP-1 failures increased during the BCx shortage, possibly driven by efforts to reduce unnecessary BCx use. Use of alternative culture sites with higher yield than blood cultures resulted in SEP-1 failures that did not result in patient harm. SEP-1 should be re-evaluated, as BCx are not the only means of diagnosing the microbiologic etiology of infection and are often low yield in select patients with sepsis.

**Figure:**
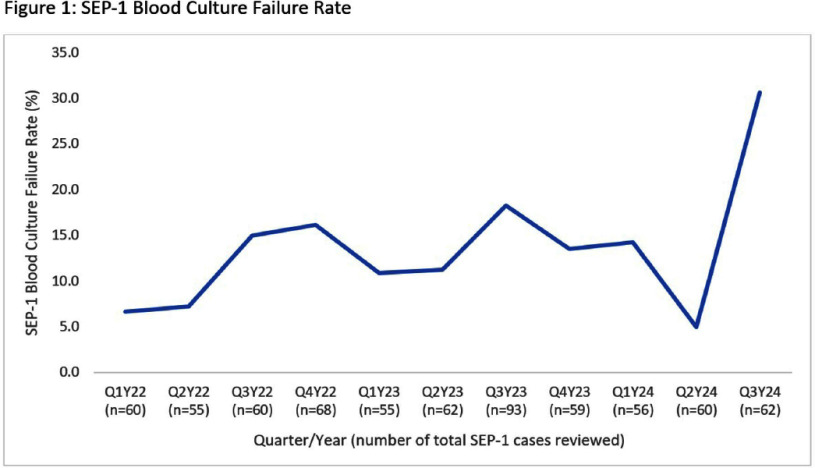

**Figure:**